# Immunopathogenesis of Human Sporotrichosis: What We Already Know

**DOI:** 10.3390/jof4030089

**Published:** 2018-07-31

**Authors:** Fatima Conceição-Silva, Fernanda Nazaré Morgado

**Affiliations:** 1 Laboratory of Immunoparasitology, Oswaldo Cruz Institute, IOC/Fiocruz, Avenida Brasil 4365 Pavilhão 26 sala 408-Manguinhos, Rio de Janeiro 21040-360, Brazil; 2 Laboratory of Leishmaniasis Research, Oswaldo Cruz Institute, IOC/Fiocruz, Avenida Brasil 4365 Pavilhão 26 sala 509-Manguinhos, Rio de Janeiro 21040-360, Brazil

**Keywords:** sporotrichosis, human, patients, immune response, immunopathogenesis, review

## Abstract

Sporotrichosis is a subacute/chronic mycosis caused by dimorphic fungus of the genus *Sporothrix.* This mycosis may affect both human and domestic animals and in the last few years, the geographic dispersion and increase of sporotrichosis worldwide has been observed. The occurrence of cases related to scratching/bites of domestic felines have increased, characterizing the disease as predominantly a zoonosis. In humans, sporotrichosis mainly involves the cutaneous tegument of infected patients, but other tissues may also present the infection. The main forms of clinical presentation are lymphocutanous sporotrichosis (LC) and fixed sporotrichosis (F). Although less common, mucosal, cutaneous disseminated, and extracutaneous forms have also been described. Multiple factors from the fungus and host can play a role in driving the clinical evolution of sporotrichosis to benign or severe disease. In this review, we discuss the immunopathological aspects involved in human sporotrichosis. Putting together the two branches of knowledge—host immune response and fungal evading mechanisms—we may perceive new possibilities in understanding the fungus–host interaction in order to be in a position to go further in the control of sporotrichosis.

## 1. The Global Increase of Sporotrichosis Creates New Problems as Well as Knowledge Opportunities

Sporotrichosis is an implantation mycosis with a subacute/chronic course caused by a dimorphic fungus of the genus *Sporothrix*. This mycosis may affect both humans and domestic animals and in the few last years, studies focused on case reports or case-series have demonstrated the geographic dispersion and the increase of sporotrichosis worldwide [1,2,3,4,5,6,7,8,9,10,11,12]. Since this mycosis is transmitted classically by traumatic inoculation through soil, vegetables, and wood containing propagules of the fungus of the genus *Sporothrix* associated with the fact that the mechanization of rural work tends to ward off these sources of infection, the increase of sporotrichosis in dense urban areas suggests changes in the epidemiological aspects [13]. Some hypotheses have been discussed in the literature, such as (i) climate changes with rises in temperature and humidity favoring fungal growth [14,15]; and (ii) the increased description of domestic animals (mainly dogs and cats) affected by sporotrichosis and implicated as dispersers of fungi in the environment and domestic space [16,17,18,19,20,21].

Faced with these new sources of infection and forms of transmission, physicians, veterinarians, and scientists now have new challenges in controlling infection in humans and animals. How can they perform differential diagnosis with other dermal agents when the fungus has not been identified? How can they evaluate the response to treatment? How do they conduct complicated human cases and those presenting atypical clinical manifestations such as the ones described in recent years? [20,22,23,24,25,26,27,28,29,30,31,32]. Although the increase in the number of cases has been accompanied by problems for the health system, it has created opportunities to produce knowledge and to deepen the study of human and animal cases that were previously scarce. In this context, a variety of previously non-existent or unaware information has emerged in recent years such as studies focused on the understanding of the higher susceptibility of cats aimed at controlling the infection in these animals [1,11,21,33]; the description of the circulation of new species and complexity in some geographic areas [10,33,34,35,36,37,38]; studies about the mechanisms involved in infection and the establishment of lesions in humans and animals by trying to understand the dynamic of fungus–vertebrate interaction [35,39,40,41,42,43,44,45]; the evaluation of new and old drugs for the treatment and resistance of some isolates have been initiated [41,46,47,48,49] as well as some correlation attempts between the susceptibility and severity of disease [50,51]. However, much more needs to be done.

## 2. Changes in the Epidemiology of Sporotrichosis Infection Alter Both, the Transmission and the Pathogenesis of the Mycosis

The *Sporothrix* complex is composed of dermophilic fungi presenting a saprophytic phase with characteristics of mycelium classically found in soil and decomposing plants from where it can be maintained by in vitro cultures at 25–26 °C. When infecting mammalian hosts, it presents a yeast-like phase, which can also be obtained in culture at 36–37 °C [52]. Several mammals such as rodents, dogs, felines (including domestic cats), and humans are susceptible to this fungal infection and it is not possible to rule out infection in other animals including wild ones, although it is difficult to detect in the latter. Classically, infection occurs by traumatic inoculation into the skin through wounds produced by spines, barbs, etc. Up to the early 2000s, only some scattered reports have described the possibility of infection from traumatic inoculation through the scratches and bites of infected animals, notably cats, but nowadays there are many descriptions [1,3,11,12,20,23,47,52,53,54,55,56,57,58,59]. Some authors have also described the possibility of infection through secretions, mainly between animals [56] and it has also been suggested that in patients presenting preexistent lesions, the transmission could also occur through licking (mainly by contaminated saliva from cats) or a contaminated environment. The lesion of nasal fossae has also been described in humans, suggesting the possibility of implantation by inhalation [58]. Consequently, the epidemiology of sporotrichosis has changed over the last 20 years. Previously considered a work-linked mycosis (farmers, gardeners, etc.) and therefore predominantly in adult males, since the late 1990s, the occurrence of cases related to the scratching/bites of domestic felines has increased, especially in Brazil, characterizing the disease as predominantly a zoonosis [1,3,11,12,20,23,47,53,54,55,56,57,58,59]. In this country, the predominant etiological agent in cats is *S. brasiliensis*, whose habitat has shifted from plants to cats, leading to epidemic sporotrichosis driven by a pathogen with a low genetic diversity [16].

In Brazil, the increased description of a zoonotic profile was initially suggested because of the reports of a large number of cases in veterinarians and veterinary workers who became infected from caring for sick animals [1,20,54,55,57,58,59]. This same characteristic has also led to an increase of in-house infection and the profile of the infected individuals has become more diverse, often including women and children, who usually have greater contact with animals.

## 3. Clinical Presentation of the Human Sporotrichosis

In humans, sporotrichosis is described as a subacute/chronic mycosis, mainly involving the cutaneous tegument of infected patients, but other tissues may also present the infection [11,12,22,24,30,47,55,56,57,58,59,60,61,62,63,64,65,66,67,68,69,70,71,72,73,74,75,76,77,78].

**Figure 1:**
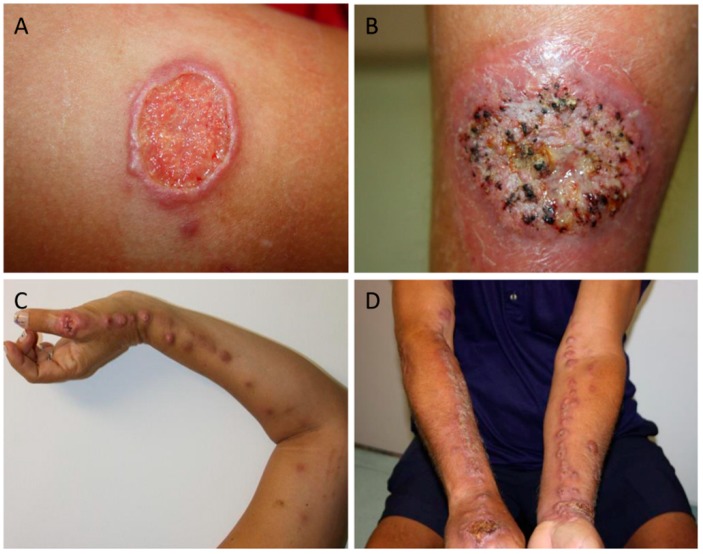
The clinical presentations of patients with the (**A**,**B**) fixed form of sporotrichosis, and (**C**,**D**) lymphocutaneous form of sporotrichosis as described in Table 1. Pictures were kindly provided by Dr. Marcelo Lyra and LapClin VigiLeish—INI-Fiocruz-RJ-Brazil.

The main forms of clinical presentation are lymphocutanous sporotrichosis (LC) and fixed sporotrichosis (F) (Figure 1). Although less common, mucosal, cutaneous disseminated, and extracutaneous forms (including osteoarticular, pulmonary, meningitis, exogenous, and endogenous endophthalmitis) have also been described (Table 1) [11,12,22,24,30,47,55,56,57,58,59,60,61,62,63,64,65,66,67,68,69,70,71,72,73,74,75,76,77,78].

The LC form is predominant in all age groups, accounting for 60% to 80% of cases in transmission areas. Approximately 25% of patients present F-forms and 5% to 10% of the cases are characterized by more severe diseases including mucosal, disseminated, and extracutaneous (pulmonary, osteoarticular, etc.) forms. Reactional cases with the presence of erythema multiforme or erythema nodosum are considered uncommon [11,30,77,78].

Depending on the geographical area where the transmission occurs, other skin and mucosal lesions such as cutaneous leishmaniasis, tuberculosis, and leprosy as well as some neoplastic and bacterial lesions should be taken into account during diagnosis since they may present a similar clinical presentation, making it difficult to diagnose when specific isolation is difficult due to technical problems. For example, in the state of Rio de Janeiro, Brazil, where the areas of transmission of sporotrichosis and American tegumentary leishmaniasis (protozoosis caused by species of the genus *Leishmania*) overlap, the presence of single ulcerated lesions may lead to confusion, moreover, the cutaneous form of sporotrichosis might be mistaken for the sporotrichoid form of ATL [79,80] since they can present very similar lesions. In these cases, the epidemiological history associated with a specific diagnostic test, mainly the isolation of the etiological agent, are fundamental in the diagnostic configuration.

Conventional treatment is achieved with itraconazole at a dose of 100 mg/day for 3–6 months. However, high dosages or longer treatment duration may be necessary [58]. Cases of resistance to the first drug of choice have been reported, therefore, the use of second choice drugs or drug association has been recommended [11,53,58,81,82].

In recent years, the possibility of different clinical presentations caused by individual differences in the host has been discussed, especially regarding the specific immune response. Circulation of cryptic species has also suggested as capable of influencing the clinical presentation of sporotrichosis in patients and animals [3,10,12].

## 4. Is the Etiological Agent a Source of Differences in Clinical Presentation?

The possible role of fungus characteristics in the disease outcome is discussed below and has been vastly discussed in other articles and reviews [3,12,52,81,83,84,85]. However, some aspects can be highlighted as different species of the genus *Sporothrix* having different grades of virulence; the role of melanine, glycoproteins, and other cell wall components as markers of virulence, among others. Recently, an increased number of evidence has pointed out the possibility of different *Sporothrix* species being capable of developing different grades of virulence based on their capacity to evade immune recognition [83], be recognized by human mononuclear cells [86], and induce the immune response [85]. In this sense, a variety of *Sporothrix* species, in the past generically characterized as *S. schenckii*, have been demonstrated in recent years. Nowadays, there is a consensus that the *S. schenckii* complex is formed by *S. schenckii*, *S. globosa*, *S. mexicana*, *S. luiriei,* and *S. brasiliensis* [10,34,87,88]. *S. brasiliensis* has been described as the most common species in Brazil where most zoonotic epidemics occur [1,53,87]. In addition, *S. brasiliensis* seems to be more virulent than *S. schenckii* since it presents a higher expression of melanin and urease [84]. In Venezuela, *S. schenckii* and *S. globosa* are the most common species that have been isolated from human lesions, and *S. globosa* has been associated with fixed lesions which represent the less severe clinical form [89]. However, Fischman Gompertz et al. evidenced atypical clinical manifestation due to *S. globosa* infection and resistance to treatment with itraconazole [90]. Regarding virulence factors, Hernández-Chaves et al. pointed out that the cell wall is a dynamic organelle vital to different functions such as cell viability, morphogenesis, and pathogenesis and has an impressive capability to adapt its composition and organization under environmental pressure. Therefore, it allows the fungus to evade immune recognition, and influences the mounting of an efficient immune response [83]. On the other hand, a better understanding of the metabolic routes, macromolecules from the cell wall, and their role in fungal survival and virulence can lead to the development of new approaches to the design of drugs and vaccines [12]. One hot field of knowledge is to understand the entrance of fungus in mammal tissues and cells as well as the first moment of interaction between the etiological agent and the host innate immune response. Despite the studies showing differences in virulence according to the *Sporothrix* species involved [91], a direct correlation between human disease and virulence profile is not conclusive [84]. For example, severe clinical presentations as disseminated forms can be observed in both immunocompetent and immunocompromised patients [25,68]. This may signal the possibility that multiple factors from the fungus and host can play a role in driving the clinical evolution of sporotrichosis to benign or severe disease. 

## 5. Immune Response in Human Sporotrichosis

One important point to highlight is the comprehension of the fungus–immune host system interaction, even in human and animals that consider the development of: (i) typical and atypical forms, (ii) benign or severe cases, and (iii) cases of easy and difficulty treatment. In this sense, some information is available. In the murine model of experimental infection, the role of cellular immunity in infection resistance has been suggested [92]. A well-modulated cellular immune response is important in order to control the infection, and an exacerbated effector function could provoke or intensify the tissue damage as observed in human mucosal lesions [93].

Data regarding the innate immune response have indicated its role in addressing the adaptive immune response. Negrini et al. demonstrated the importance of TLR2 at the beginning of the immune response once this receptor facilitates the phagocytosis of fungal elements stimulating the production of cytokines such as TNF-α, IL-12, IL-1β, IL-10, IFN-γ, IL-6, IL-17, and TGF-β as well as nitric oxide [45]. These cytokines have been implicated in the differentiation of T lymphocytes into Th1 and Th17 cells, and in the regulation of effector functions in specific anti-fungal response [94]. Other components of the in situ immune response, notably in the innate-adaptive response interface, may play an important role in the differentiation of T cells and immunological effectors. For example, dendritic cells (DC) may regulate the pro-inflammatory response to fungal elements depending on the recognition and quantity of antigens [44]. The authors evidenced that antigens in higher concentrations may stimulate the expression of cytokines IL-23, IL-6, TGF-β, and IL-17, consequently leading to the differentiation of T cells into Th1 and Th17 in vitro suggesting that different initial quantities of stimuli may induce different immune responses [44]. In the murine model, Th1 and Th17 cells are induced during *S. schenckii* infection; however, intact Th17 cells are needed for the clearance of the fungal load [95]. Even in the absence of Th1 cells, Th17 cells were able to control parasite load and treatment with the anti-IL-23 antibody impaired the ability to control fungal replication [95]. In humans, even in established lesions, the abundance of neutrophils was accompanied by a higher parasite load and characterized the most exuberant clinical forms [40]. The suppurative inflammatory reaction associated with neutrophil infiltration was also demonstrated in lymphocutaneous lesions [96]. The association between Th17 cells and neutrophils is well-described in fungal infections [97]; however, it is not the unique cell cooperation described in mycoses. In this sense, in a murine model of experimental candidiasis, Th17 cells were crucial to the control of fungal load and was associated with mature NK cells and the NK cells’ capacity to kill target cells, produce IFN-γ, and promote the activity of neutrophils through GM-CSF secretion [98]. In another study, the reduction of IL-17 was accompanied by the increase of fungal load in mice experimentally infected with *S. schenckii* [99]. Dectin-1 and dectin-2 are cell receptors involved in the recognition and the immune response against fungus [100,101]. Dectin-1 and dectin-2 recognize fungal surface sugar polymers such as β-glucan and α-mannan, respectively [102]. Although in different pathways, both recognitions induce the differentiation of Th17 cells, reactive oxygen species, and the expression of pro-inflammatory cytokines in *Candida albicans* and other fungal infections [101,103]. In mice experimentally infected with *S. schenckii*, there was an association between the increase of dectin-1 expression by macrophages and nitric oxide, IL-10, IL-1β, and TNF [104]. However, in human sporotrichosis, these details need to be clarified and a better characterization of neutrophil function and enzymes associated with the control of fungal burden may help in understanding both the infection and lesion development dynamics.

Mast cells are tissue resident cells observed all over the body. Their role in sporotrichosis has been shown in vitro and in vivo in the murine model [43]. In this study, stimulated mast cells with conidia from *S. schenckii* were capable of degranulating to release histamine and produce TNF and IL-6, thus playing a role in the initial neutrophil influx that participates in the conversion from mycelial to yeast form [43]. In vivo, mast cells contribute to fungal dissemination and disease worsening [43].

Although excellent, the murine model may not entirely reproduce the factors regarding the development of different clinical forms as observed in human sporotrichosis. Due to the shortage in classic cases, the available information about the specific immune response to *S. schenckii* by patients is inconsistent, mainly related to the in situ inflammation data, a field where few studies have been published [40,80,96,105,106,107]. In two of the first studies published, five cases were evaluated and the authors showed that 0.2–0.8% of cells in inflammatory infiltration were dendritic cells [105,106]. They also detected the infiltration of CD4 and CD8 T cells as well as IFN-γ expressing cells, similar to mononuclear lymphoid cells in the periphery of granulomas [105,106]. In two other papers, skin lesions of human sporotrichosis were evaluated by histopathological analysis [96] and the composition/organization of inflammatory infiltration were evaluated through immunohistochemistry [40]. In the last one, fixed lesions were compared to lymphocutaneous lesions, and the latter showed more extension and severity than fixed lesions due to more intense inflammatory infiltrates, leading to more intense tissue destruction. On the other hand, fixed lesions showed a more balanced and efficient immune response leading to the control of infection without the destruction of adjacent tissue and fungal dissemination [40,80]. Lymphocutaneous skin lesions presented more intense and diffuse inflammatory infiltrates associated with necrosis and suppurative reactions, characterizing an unbalanced immune response [80]. A higher severity was associated with a higher percentage of CD4 T cells, CD22 B cells, neutrophils, and NOS2 expression, leading to more intense inflammatory activity. Similar to human skin lesions, in the experimental infection of the murine model, a cellular immune response dependent on nitric oxide was observed as well as a Th2 and humoral immune response in advanced stages [108]. The treatment with anti-*S. schenckii* antibodies has led to the reduction of parasite load in various organs in mice [109]. These data together suggest that host immune response, mainly that produced by the skin, maintains a phenotypic and functional pattern regardless of the fact that some peculiarities influence disease progression. In fact, there is a consensus that the cellular immune response is essential to control fungal infection, but immunoglobulins from Th1 response also play a beneficial role [110]. The enhancement of macrophage ability to phagocytize opsonized fungal elements by immunoglobulins has reinforced this hypothesis [41,111]. In addition, in higher IL-1β and TNF expression, pro-inflammatory cytokines related to macrophage activation were observed [111]. In Balb/c mice, the reduction of IL-1β, caspase-1, and IL-18 coincided with the immunosuppression transitory stage and increase of fungal load, suggesting the role of inflammasome in anti-*S. schenckii* response [99]. Maia et al. also observed the relation of IL-1β in the early control of fungal load, but in addition to H2O2 and IL-2 [112]. In fact, knockout (KO) mice NLRP3^−/−^, ASC^−/−^, and caspase-1^−/−^ were more susceptible to *S. schenckii* infection than wild-type animals [113]. Furthermore, KO mice showed reduced Th17 and Th1 differentiation and reduced production of IL-17 and IL-8, but favored Treg cell differentiation [113].

Fungus can use components of the host immune effectors to survive [83]. In this sense, asteroid bodies are characterized as spherical yeasts covered by concentric layers of deposited material such as IgG and IgM [114]. The deposited material confers fungal resistance to specific immune response, protecting fungal antigens, and maintaining fungal viability and survival [114]. Furthermore, some fungal compounds are able to influence host immune response, pathogenicity, and invasion [112,115]. Rodrigues et al. identified gp70, a cell wall compound which consists of an adhesion molecule for fibronectin and laminin and is able to induce a strong humoral response in patients from different clinical forms [115]. Different species seem to induce different host immune responses due to cell wall composition. In this context, *S. schenckii* and *S. brasiliensis* are differentially recognized by human PBMCs [86]: *S. brasiliensis* induced higher IL-10 expression and lower TNF and IL-6 expression, and IL-10 expression was dependent on dectin-1 recognition by human cells [86]. Some of the identified cell wall compounds have been evaluated as vaccine candidates [85,116]. Anti-cell wall compound sera from immunized mice was able to induce phagocytosis and inhibit the adhesion of the fungus to fibroblasts [116,117]. Furthermore, the immunization induced IFN-γ, IL-4, IL-17, and IL-12 expression [116,117]. Gp70 was also evaluated as a vaccine candidate and induced strong cellular (Th1/Th17) and humoral response in mice immunized with recombinant phages [118]. The protection was confirmed after challenge and mice immunized with recombinant phages showed lower CFU and inflammatory infiltration [118]. In another study, the ZR8 peptide from gp70 induced CD4 T cells and higher levels of INF-γ, IL-1β, and IL-17A as well as a higher number of neutrophils in skin lesions after challenge, which are associated with fungal control [119].

Concerning the role of nitric oxide, the data available are controversial. Nitric oxide production is induced by conidia and yeast cells and has been described as a fungicidal molecule to *S. schenckii* in vitro [120]. However, in the experimental infection of the wild type and NOS2^−/−^ murine model, NO induced T cell suppression and fungal dissemination, leading to the death of wild type animals [121]. This data suggested a deleterious effect of NO in vivo when *S. schenckii* experimentally infected animals were evaluated. In fact, in skin lesions from sporotrichosis patients (humans), NOS2 expression was associated with more extended and severe lesions such as the lymphocutaneous form as well as to higher fungal load, suggesting that NO may induce tissue damage favoring fungal spread [40,80]. In addition, lymphocutaneous lesions presented higher IL-10 expression probably as a regulatory mechanism that compensates skin damage, however favoring the escape of fungus from host defense effectors [107]. IL-10 as well as arginase-1 and TGFβ can be produced by alternatively activated macrophages (M2), which are cells involved in tissue remodeling, angiogenesis, and repair [122], however, are unable to express NOS2 and kill fungal cells serving as a site for parasite survival. M2 cells can be stimulated by cell wall peptide-polysaccharides from *S. schenckii* in a murine model of infection [122].

Fernandes et al. suggested a direct association between virulence and resistance to NO [120]. The presence of melanin was also associated with pathogenicity [12,123]. Rats infected with a melanin positive isolate presented a higher frequency of lesions, lymphatic alterations, and lymphadenopathy than animals infected with the melanin negative mutant strain [123]. Upon histopathological analysis, the melanin positive isolates induced multiple granulomas, while the mutant strain induced focal and restricted granulomatous reaction [123]. The authors considered that melanin protects the fungus from macrophage phagocytosis and from oxygen and nitrogen oxidative radicals [123]. In this context, the presence of fungus in the host tissue stimulates an inflammatory reaction with granuloma formation, IFN-γ, and NOS2 expression, leading to tissue damage. Despite this hostile milieu, the fungus can resist oxidative effectors, in part due to its melanin constitution, and disseminate to other sites generating extended lesions as observed in the lymphocutaneous form.

Sporotrichosis has also been reported in immunosuppressed patients. Disseminated sporotrichosis has been described in one liver transplanted patient [124]. In co-infection HIV/*S. schenckii*, the clinical exacerbation of skin lesions or unusual clinical forms such as cutaneous dissemination, extracutaneous forms, or the development of meningitis in some cases has been observed as a result of immune reconstitution inflammatory syndrome [22,71,73,74,75]. Some patients have shown skin lesions before central nervous system involvement, which suggests the hematogenous dissemination of fungus [74]. Two systematic reviews focusing on HIV/*S. schenckii* co-infection have been published [70,72] where the authors showed that most clinical forms were disseminated and cutaneous disseminated. The lower CD4 T cell numbers were associated with the disseminated form, high mortality, and a correlation between the involvement of the central nervous system and death [70]. They also observed unusual manifestations such as meningitis, endophtalmitis, primary pulmonary disease, endocarditis, primary sinus disease, and IRIS [70,72]. In a systematic review of endophthalmitis caused by *Sporothrix* species, the authors described two basic types: (1) the endogenous endophthalmitis produced by fungus dissemination all over the body and more common in HIV patients and those ones living in hyperendemic areas; and (2) exogenous endophthalmitis caused by direct traumatic inoculation [72]. In a case report, a correlation between osteoarticular sporotrichosis after hematogenous spread and alcohol abuse was described [125]. In a review paper, the authors discussed the cellular and molecular defects of the immune response that were predisposed to systemic fungal infections such as those observed in the co-infected HIV/*S. schenckii* patients [126]. Although rare, primary pulmonary sporotrichosis has been also described, though mainly in patients with a long history of smoking [76].

An increase in cases of drug resistance depending on the fungal species has also been shown [1,35,45,88,106]. Almeida-Paes et al. observed an *S. schenckii* isolate resistant to terbinafine and attributed this resistance to melanin protection [127]. In a recent work, the impact of repeated exposure to mercury (Hg) was evaluated in infected mice [128]. Treatment with HgCl_2_ impaired the immune response, affecting the production of IFN-γ, IL-1, and NO by macrophages, Th1/Th2/Th17 quantities, and their respective cytokines, suggesting that repeated exposure to mercury such as those observed in Hg-polluted areas enhanced susceptibility to *Sporothrix* infection and could be associated with sporotrichosis outbreaks [128]. Based on these data, we can hypothesize that good or bad treatment response may be determined by both the fungal agent and the host immune response capability to control fungal replication and dissemination. This hypothesis can be supported by the results obtained in a murine model of experimental infection [88] and by the evaluation of human cases [46]. Together with the increasing numbers of sporotrichosis cases, mainly in previously safe areas, these facts challenge health professionals to confirm and treat the cases of disease. Furthermore, they face the difficulty of clinical diagnosis due to clinical similarities with several other infectious skin or non-skin lesions [28,32,129,130,131,132,133,134,135,136,137]. For example, in a comparative study on the immunopathology of tegumentary leishmaniasis and sporotrichosis, the lesions were macroscopically very similar, however microscopically, they differed in quantities of neutrophils, macrophages, CD8 T cells, CD4 T cells, NOS2, B cells, and FasL+ cells [80].

These data suggest that the skin immune system is a complex, adaptable system capable of different responses to intracellular or extracellular pathogens [80] and that the composition of the inflammatory infiltrates may be used to differentiate lesions of sporotrichosis from tegumentary leishmaniasis when the identification of the etiological agent cannot be undertaken. However, data associated with the study of the immune response from human patients are still scarce in the literature and should be further explored with the aim to elucidate the immunological mechanisms that determine the development of lesions and the response to treatment.

## 6. Conclusions

The increased number of cases of human sporotrichosis together with the worldwide spread observed in the last few years, has led physicians and veterinarians to face new challenges in the diagnosis, treatment, and monitoring of a not well known mycosis. On the other hand, it has also led to an opportunity to deepen the information and knowledge about the disease and its etiological agent. Knowledge has been increased over the past 18 years including the description/detailing of the *Sporothrix* complex as well as the suggestion of the role of both innate and adaptative immune response as playing a role in the clinical presentation and treatment response of human sporotrichosis. However, several missing pieces of information still need to be elucidated to improve the understanding of sporotrichosis pathogenesis. Placing together the two branches of knowledge—host immune response and fungal evading mechanisms—we may perceive new possibilities in understanding the interaction fungus—host to be able to go further in the control of Sporotrichosis.

## Figures and Tables

**Table 1:** Clinical presentation of human sporotrichosis.

Clinical Presentation	Main Features	Some Reports
Fixed	Single nodule, ulcer, or verrucous lesion without signs of lymphangitis	[11,12,47,53,55,56,57,58,59]
Lymphocutaneous	Multiple nodules, ulcers, and signs of lymphangitis following the path of lymphatic draining	[11,12,47,53,55,56,57,58,59]
Mucosal	Ulcers, granulomatous infiltration, serous-purulent discharge, crusts in conjunctiva, nasal and/or oral mucosa	[11,12,47,53,55,56,57]
Cutaneous disseminated in immunocompetent or immunocompromised patients	Multiple nodules and/or ulcers in different non-contiguous parts of the body	[11,12,47,53,55,56,57,58,59]
Extracutaneous in immunocompetent or immunocompromised patients	Osteoarticular, pulmonary, meningitis, exogenous and endogenous endophthalmitis etc.	[22,24,30,57,60,61,62,63,64,65,66,67,68,69,70,71,72,73,74,75,76]
Reactional cases	Erythema multiforme, erythema nodosum	[30,77,78]

## References

[1] Gremião I.D.F., Miranda L.H.M., Reis E.G., Rodrigues A.M., Pereira S.A. (2017). Zoonotic Epidemic of Sporotrichosis: Cat to Human Transmission. PLoS Pathog..

[2] Chakrabarti A., Bonifaz A., Gutierrez-Galhardo M.C., Mochizuki T., Li S. (2015). Global epidemiology of sporotrichosis. Med. Mycol..

[3] López-Romero E., Reyes-Montes M.D., Pérez-Torres A., Ruiz-Baca E., Villagómez-Castro J.C., Mora-Montes H.M., Flores-Carreón A., Toriello C. (2011). *Sporothrix schenckii* complex and sporotrichosis, an emerging health problem. Future Microbiol..

[4] Bhutia P.Y., Gurung S., Yegneswaran P.P., Pradhan J., Pradhan U., Peggy T., Pradhan P.K., Bhutia C.D. (2011). A case series and review of sporotrichosis in Sikkim. J. Infect. Dev. Ctries..

[5] Dias N.M., Oliveira M.M.E., Santos C., Zancope-Oliveira R.M., Lima N. (2011). Sporotrichosis caused by *Sporothrix mexicana*, Portugal. Emerg. Infect. Dis..

[6] Song Y., Yao L., Zhong S.-X., Tian Y.-P., Liu Y.-Y., Li S.-S. (2011). Infant sporotrichosis in northeast China: A report of 15 cases. Int. J. Dermatol..

[7] Sivagnanam S., Bannan A.M., Chen S.C.-A., Ralph A.P. (2012). Sporotrichosis (*Sporothrix schenckii* infection) in the New South Wales mid-north coast, 2000–2010. Med. J. Aust..

[8] Verma S., Verma G.K., Singh G., Kanga A., Shanker V., Singh D., Gupta P., Mokta K., Sharma V. (2012). Sporotrichosis in sub-himalayan India. PLoS Negl. Trop. Dis..

[9] Mata-Essayag S., Delgado A., Colella M.T., Landaeta-Nezer M.E., Rosello A., Perez de Salazar C., Olaizola C., Hartung C., Magaldi S., Velasquez E. (2013). Epidemiology of sporotrichosis in Venezuela. Int. J. Dermatol..

[10] Oliveira M.M.E., Almeida-Paes R., Gutierrez-Galhardo M.C., Zancope-Oliveira R.M. (2014). Molecular identification of the *Sporothrix schenckii* complex. Rev. Iberoam. Micol..

[11] De Lima Barros M.B., de Almeida Paes R., Schubach A.O. (2011). *Sporothrix schenckii* and Sporotrichosis. Clin. Microbiol. Rev..

[12] Lopes-Bezerra L.M., Mora-Montes H.M., Zhang Y., Nino-Veja G., Rodrigues A.M., de Camargo Z.P., de Hoog S. (2018). Sporotrichosis between 1898 and 2017: The evolution of knowledge on a changeable disease and on emerging etiological agents. Med. Mycol..

[13] Sanchotene K.O., Madrid I.M., Klafke G.B., Bergamashi M., Della Terra P.P., Rodrigues A.M., de Camargo Z.P., Xavier M.O. (2015). *Sporothrix brasiliensis* outbreaks and the rapid emergence of feline sporotrichosis. Mycoses.

[14] Téllez M.D., Batista-Duharte A., Portuondo D., Quinello C., Bonne-Hernández R., Carlos I.Z. (2014). *Sporothrix schenckii* complex biology: Environment and fungal pathogenicity. Microbiology.

[15] Rangel-Gamboa L., Martínez-Hernandez F., Maravilla P., Arenas-Guzmán R., Flisser A. (2016). Update of phylogenetic and genetic diversity of *Sporothrix schenckii* sensu lato. Med. Mycol..

[16] Rodrigues A.M., de Hoog G.S., Zhang Y., de Camargo Z.P. (2014). Emerging sporotrichosis is driven by clonal and recombinant *Sporothrix* species. Emerg. Microbes Infect..

[17] Miranda L.H.M., Quintella L.P., Menezes R.C., dos Santos I.B., Oliveira R.V.C., Figueiredo F.B., Lopes-Bezerra L.M., Schubach T.M. (2011). Evaluation of immunohistochemistry for the diagnosis of sporotrichosis in dogs. Vet. J..

[18] Pereira S.A., Menezes R.C., Gremião I.D.F., Silva J.N., de Honse C.O., Figueiredo F.B., da Silva D.T., Kitada A.A., dos Reis E.G., Schubach T.M. (2011). Sensitivity of cytopathological examination in the diagnosis of feline sporotrichosis. J. Feline Med. Surg..

[19] Rees R.K., Swartzberg J.E. (2011). Feline-transmitted sporotrichosis: A case study from California. Dermatol. Online J..

[20] Borges T.S., Rossi C.N., Fedullo J.D.L., Taborda C.P., Taborda J.P., Larsson C.E. (2013). Isolation of *Sporothrix schenckii* from the claws of domestic cats (indoor and outdoor) and in captivity in São Paulo (Brazil). Mycopathologia.

[21] Miranda L.H.M., Conceição-Silva F., Quintella L.P., Kuraiem B.P., Pereira S.A., Schubach T.M.P. (2013). Feline sporotrichosis: Histopathological profile of cutaneous lesions and their correlation with clinical presentation. Comp. Immunol. Microbiol. Infect. Dis..

[22] Bonifaz A., Tirado-Sánchez A. (2017). Cutaneous Disseminated and Extracutaneous Sporotrichosis: Current Status of a Complex Disease. J. Fungi.

[23] Costa R.O., Bernardes-Engemann A.R., Azulay-Abulafia L., Benvenuto F., de Neves M.L.P., Lopes-Bezerra L.M. (2011). Sporotrichosis in pregnancy: Case reports of 5 patients in a zoonotic epidemic in Rio de Janeiro, Brazil. An. Bras. Dermatol..

[24] Ferreira C.P., do Valle A.C.F., Freitas D.F.S., Reis R., Galhardo M.C.G. (2012). Pregnancy during a sporotrichosis epidemic in Rio de Janeiro, Brazil. Int. J. Gynaecol. Obstet..

[25] Freitas D.F.S., de Siqueira Hoagland B., do Valle A.C.F., Fraga B.B., de Barros M.B., de Oliveira Schubach A., de Almeida-Paes R., Cuzzi T., Rosalino C.M., Zancopé-Oliveira R.M. (2012). Sporotrichosis in HIV-infected patients: Report of 21 cases of endemic sporotrichosis in Rio de Janeiro, Brazil. Med. Mycol..

[26] Chang S., Hersh A.M., Naughton G., Mullins K., Fung M.A., Sharon V.R. (2013). Disseminated cutaneous sporotrichosis. Dermatol. Online J..

[27] Gewehr P., Jung B., Aquino V., Manfro R.C., Spuldaro F., Rosa R.G., Goldani L.Z. (2013). Sporotrichosis in renal transplant patients. Can. J. Infect. Dis. Med. Microbiol..

[28] Orofino-Costa R., Unterstell N., Carlos Gripp A., de Macedo P.M., Brota A., Dias E., de Melo Teixeira M., Felipe M.S., Bernardes-Engemann A.R., Lopes-Bezerra L.M. (2013). Pulmonary cavitation and skin lesions mimicking tuberculosis in a HIV negative patient caused by *Sporothrix brasiliensis*. Med. Mycol. Case Rep..

[29] Sharon V.R., Kim J., Sudhakar S., Fung M.A., Maniar A. (2013). Disseminated cutaneous sporotrichosis. Lancet Infect. Dis..

[30] Xavier M.O., Bittencourt L.R., da Silva C.M., Vieira R.S., Pereira H.C.P. (2013). Atypical presentation of sporotrichosis: Report of three cases. Rev. Soc. Bras. Med. Trop..

[31] De Carvalho Aguinaga F., Trope B.M., Fernandes N.C., Engel D.C., Ramos-E-Silva M. (2014). Sporotrichosis with bone involvement: An alert to an occupational disease. Case Rep. Dermatol..

[32] Zhang Y., Pyla V. (2014). Cancer-like lesions in a patient with sporotrichosis. Int. J. Dermatol..

[33] Miranda L.H.M., de Santiago M.A., Schubach T.M.P., Morgado F.N., Pereira S.A., de Oliveira R.V.C., Conceição-Silva F. (2016). Severe feline sporotrichosis associated with an increased population of CD8low cells and a decrease in CD4^+^ cells. Med. Mycol..

[34] Oliveira M.M.E., Almeida-Paes R., Muniz M.M., Gutierrez-Galhardo M.C., Zancope-Oliveira R.M. (2011). Phenotypic and molecular identification of *Sporothrix* isolates from an epidemic area of sporotrichosis in Brazil. Mycopathologia.

[35] Fernandes G.F., dos Santos P.O., Rodrigues A.M., Sasaki A.A., Burger E., de Camargo Z.P. (2013). Characterization of virulence profile, protein secretion and immunogenicity of different *Sporothrix schenckii* sensu stricto isolates compared with *S. globosa* and *S. brasiliensis* species. Virulence.

[36] Liu X., Zhang Z., Hou B., Wang D., Sun T., Li F., Wang H., Han S. (2013). Rapid identification of *Sporothrix schenckii* in biopsy tissue by PCR. J. Eur. Acad. Dermatol. Venereol..

[37] Oliveira M.M.E., Maifrede S.B., Ribeiro M.A., Zancope-Oliveira R.M. (2013). Molecular identification of *Sporothrix* species involved in the first familial outbreak of sporotrichosis in the state of Espírito Santo, southeastern Brazil. Mem. Inst. Oswaldo Cruz.

[38] Rodrigues A.M., de Hoog G.S., de Camargo Z.P. (2014). Genotyping species of the *Sporothrix schenckii* complex by PCR-RFLP of calmodulin. Diagn. Microbiol. Infect. Dis..

[39] Tachibana T., Matsuyama T., Mitsuyama M. (1998). Characteristic infectivity of *Sporothrix schenckii* to mice depending on routes of infection and inherent fungal pathogenicity. Med. Mycol..

[40] Morgado F.N., Schubach A.O., Barros M.B.L., Conceição-Silva F. (2011). The in situ inflammatory profile of lymphocutaneous and fixed forms of human sporotrichosis. Med. Mycol..

[41] Guzman-Beltran S.L., Perez-Torres A., Coronel-Cruz C., Torres-Guerrero H. (2012). Phagocytic receptors on macrophages distinguish between different *Sporothrix schenckii* morphotypes. Microbes Infect..

[42] Li M., Chen Q., Sun J., Shen Y., Liu W. (2012). Inflammatory response of human keratinocytes triggered by *Sporothrix schenckii* via Toll-like receptor 2 and 4. J. Dermatol. Sci..

[43] Romo-Lozano Y., Hernández-Hernández F., Salinas E. (2012). Mast cell activation by conidia of *Sporothrix schenckii*: Role in the severity of infection. Scand. J. Immunol..

[44] Verdan F.F., Faleiros J.C., Ferreira L.S., Monnazzi L.G.S., Maia D.C.G., Tansine A., Placeres M.C., Carlos I.Z., Santos-Junior R.R. (2012). Dendritic cell are able to differentially recognize *Sporothrix schenckii* antigens and promote Th1/Th17 response in vitro. Immunobiology.

[45] De Negrini T.C., Ferreira L.S., Alegranci P., Arthur R.A., Sundfeld P.P., Maia D.C.G., Spolidorio L.C., Carlos I.Z. (2013). Role of TLR-2 and fungal surface antigens on innate immune response against *Sporothrix schenckii*. Immunol. Investig..

[46] Galhardo M.C.G., De Oliveira R.M.Z., Valle A.C.F.D., Paes R.D.A., Silvatavares P.M.E., Monzon A., Mellado E., Rodriguez-Tudela J.L., Cuenca-Estrella M. (2008). Molecular epidemiology and antifungal susceptibility patterns of *Sporothrix schenckii* isolates from a cat-transmitted epidemic of sporotrichosis in Rio de Janeiro, Brazil. Med. Mycol..

[47] De Lima Barros M.B., Schubach A.O., de Vasconcellos Carvalhaes de Oliveira R., Martins E.B., Teixeira J.L., Wanke B. (2011). Treatment of cutaneous sporotrichosis with itraconazole—Study of 645 patients. Clin. Infect. Dis..

[48] Fernández-Silva F., Capilla J., Mayayo E., Guarro J. (2014). Modest efficacy of voriconazole against murine infections by *Sporothrix schenckii* and lack of efficacy against *Sporothrix brasiliensis*. Mycoses.

[49] Ottonelli Stopiglia C.D., Magagnin C.M., Castrillón M.R., Mendes S.D.C., Heidrich D., Valente P., Scroferneker M.L. (2014). Antifungal susceptibilities and identification of species of the *Sporothrix schenckii* complex isolated in Brazil. Med. Mycol..

[50] Della Terra P.P., Rodrigues A.M., Fernandes G.F., Nishikaku A.S., Burger E., de Camargo Z.P. (2017). Exploring virulence and immunogenicity in the emerging pathogen *Sporothrix brasiliensis*. PLoS Negl. Trop. Dis..

[51] Brito M.M.S., Conceição-Silva F., Morgado F.N., Raibolt P.S., Schubach A., Schubach T.P., Schäffer G.M., Borba C.M. (2007). Comparison of virulence of different *Sporothrix schenckii* clinical isolates using experimental murine model. Med. Mycol..

[52] Boyce K.J., Andrianopoulos A. (2015). Fungal dimorphism: The switch from hyphae to yeast is a specialized morphogenetic adaptation allowing colonization of a host. FEMS Microbiol. Rev..

[53] Schubach A., de Barros M.B.L., Wanke B. (2008). Epidemic sporotrichosis. Curr. Opin. Infect. Dis..

[54] Gremião I.D.F., Menezes R.C., Schubach T.M.P., Figueiredo A.B.F., Cavalcanti M.C.H., Pereira S.A. (2015). Feline sporotrichosis: Epidemiological and clinical aspects. Med. Mycol..

[55] Barros M.B.L., Schubach A.O., Schubach T.M.P., Wanke B., Lambert-Passos S.R. (2008). An epidemic of sporotrichosis in Rio de Janeiro, Brazil: Epidemiological aspects of a series of cases. Epidemiol. Infect..

[56] Lopes-Bezerra L.M., Schubach A., Costa R.O. (2006). *Sporothrix schenckii* and sporotrichosis. An. Acad. Bras. Cienc..

[57] Schubach A., Schubach T.M.P., de Barros M.B.L., Wanke B. (2005). Cat-transmitted sporotrichosis, Rio de Janeiro, Brazil. Emerg. Infect. Dis..

[58] De Barros M.B.L., de Schubach A.O., do Valle A.C.F., Gutierrez Galhardo M.C., Conceição-Silva F., Schubach T.M.P., Reis R.S., Wanke B., Marzochi K.B., Conceição M.J. (2004). Cat-transmitted sporotrichosis epidemic in Rio de Janeiro, Brazil: Description of a series of cases. Clin. Infect. Dis..

[59] De Lima Barros M.B., Schubach T.M., Galhardo M.C., de Oliviera Schubach A., Monteiro P.C., Reis R.S., Zancopé-Oliveira R.M., dos Santos Lazéra M., Cuzzi-Maya T., Blanco T.C. (2001). Sporotrichosis: An emergent zoonosis in Rio de Janeiro. Mem. Inst. Oswaldo Cruz.

[60] Baum G.L., Donnerberg R.L., Stewart D., Mulligan W.J., Putnam L.R. (1969). Pulmonary sporotrichosis. N. Engl. J. Med..

[61] Smith A.G., Morgan W.K., Hornick R.B., Funk A.M. (1970). Chronic pulmonary sporotrichosis: Report of a case, including morphologic and mycologic studies. Am. J. Clin. Pathol..

[62] England D.M., Hochholzer L. (1985). Primary pulmonary sporotrichosis. Report of eight cases with clinicopathologic review. Am. J. Surg. Pathol..

[63] Donabedian H., O’Donnell E., Olszewski C., MacArthur R.D., Budd N. (1994). Disseminated cutaneous and meningeal sporotrichosis in an AIDS patient. Diagn. Microbiol. Infect. Dis..

[64] Khabie N., Boyce T.G., Roberts G.D., Thompson D.M. (2003). Laryngeal sporotrichosis causing stridor in a young child. Int. J. Pediatr. Otorhinolaryngol..

[65] Silva-Vergara M.L., Maneira F.R.Z., De Oliveira R.M., Santos C.T.B., Etchebehere R.M., Adad S.J. (2005). Multifocal sporotrichosis with meningeal involvement in a patient with AIDS. Med. Mycol..

[66] Appenzeller S., Amaral T.N., Amstalden E.M.I., Bertolo M.B., Neto J.F.M., Samara A.M., Fernandes S.R. (2006). *Sporothrix schenckii* infection presented as monoarthritis: Report of two cases and review of the literature. Clin. Rheumatol..

[67] Aung A.K., Spelman D.W., Thompson P.J. (2015). Pulmonary Sporotrichosis: An Evolving Clinical Paradigm. Semin. Respir. Crit. Care Med..

[68] Freitas D.F.S., Santos S.S., Almeida-Paes R., de Oliveira M.M.E., do Valle A.C.F., Gutierrez-Galhardo M.C., Zancopé-Oliveira R.M., Nosanchuk J.D. (2015). Increase in virulence of *Sporothrix brasiliensis* over five years in a patient with chronic disseminated sporotrichosis. Virulence.

[69] Freitas D.F.S., Lima M.A., de Almeida-Paes R., Lamas C.C., do Valle A.C.F., Oliveira M.M.E., Zancopé-Oliveira R.M., Gutierrez-Galhardo M.C. (2015). Sporotrichosis in the Central Nervous System Caused by *Sporothrix brasiliensis*. Clin. Infect. Dis..

[70] Moreira J.A.S., Freitas D.F.S., Lamas C.C. (2015). The impact of sporotrichosis in HIV-infected patients: A systematic review. Infection.

[71] Biancardi A.L., Freitas D.F.S., Vitor R.D.A., Andrade H.B., de Oliveira M.M.E., do Valle A.C.F., Zancope-Oliveira R.M., Galhardo M.C., Curi A.L. (2017). Multifocal choroiditis in disseminated sporotrichosis in patients with HIV/AIDS. Retin. Cases Brief Rep..

[72] Ramírez Soto M.C. (2018). Differences in clinical ocular outcomes between exogenous and endogenous endophthalmitis caused by *Sporothrix*: A systematic review of published literature. Br. J. Ophthalmol..

[73] Paixão A.G., Galhardo M.C.G., Almeida-Paes R., Nunes E.P., Gonçalves M.L.C., Chequer G.L., Lamas C.D.C. (2015). The difficult management of disseminated *Sporothrix brasiliensis* in a patient with advanced AIDS. AIDS Res. Ther..

[74] Galhardo M.C.G., Silva M.T.T., Lima M.A., Nunes E.P., Schettini L.E.C., de Freitas R.F., de Paes R.A., de Neves E.S., do Valle A.C. (2010). *Sporothrix schenckii* meningitis in AIDS during immune reconstitution syndrome. J. Neurol. Neurosurg. Psychiatry.

[75] Lyra M.R., Nascimento M.L.F.O., Varon A.G., Pimentel M.I.F., de Antonio L.F., Saheki M.N., Bedoya-Pacheco S.J., Valle A.C. (2014). Immune reconstitution inflammatory syndrome in HIV and sporotrichosis coinfection: Report of two cases and review of the literature. Rev. Soc. Bras. Med. Trop..

[76] Rojas F.D., Fernández M.S., Lucchelli J.M., Lombardi D., Malet J., Vetrisano M.E., Cattana M.E., Sosa M.L.Á., Giusiano G. (2017). Cavitary Pulmonary Sporotrichosis: Case Report and Literature Review. Mycopathologia.

[77] Gutierrez Galhardo M.C., de Oliveira Schubach A., de Lima Barros M.B., Moita Blanco T.C., Cuzzi-Maya T., Pacheco Schubach T.M., dos Santos Lazéra M., do Valle A.C. (2002). Erythema nodosum associated with sporotrichosis. Int. J. Dermatol..

[78] Papaiordanou F., da Silveira B.R.L., Abulafia L.A. (2015). Hypersensitivity reaction to Sporothrix schenckii: Erythema nodosum associated with sporotrichosis. Rev. Soc. Bras. Med. Trop..

[79] De Carvalho L.M.V., Pimentel M.I.F., Conceição-Silva F., Valete-Rosalino C.M., Lyra M.R., Salgueiro M.M., Saheki M.N., Madeira M.F., Mouta-Confort E., Antonio L.F. (2017). Sporotrichoid leishmaniasis: A cross-sectional clinical, epidemiological and laboratory study in Rio de Janeiro State, Brazil. Rev. Inst. Med. Trop. São Paulo.

[80] Morgado F.N., de Carvalho L.M.V., Leite-Silva J., Seba A.J., Pimentel M.I.F., Fagundes A., Madeira M.F., Lyra M.R., Oliveira M.M., Schubach A.O. (2018). Unbalanced inflammatory reaction could increase tissue destruction and worsen skin infectious diseases—A comparative study of leishmaniasis and sporotrichosis. Sci. Rep..

[81] Almeida-Paes R., Oliveira M.M.E., Freitas D.F.S., do Valle A.C.F., Gutierrez-Galhardo M.C., Zancopé-Oliveira R.M. (2017). Refractory sporotrichosis due to *Sporothrix brasiliensis* in humans appears to be unrelated to in vivo resistance. Med. Mycol..

[82] Almeida-Paes R., Brito-Santos F., Figueiredo-Carvalho M.H.G., Machado A.C.S., Oliveira M.M.E., Pereira S.A., Gutierrez-Galhardo M.C., Zancopé-Oliveira R.M. (2017). Minimal inhibitory concentration distributions and epidemiological cutoff values of five antifungal agents against *Sporothrix brasiliensis*. Mem. Inst. Oswaldo Cruz.

[83] Hernández-Chávez M.J., Pérez-García L.A., Niño-Vega G.A., Mora-Montes H.M. (2017). Fungal Strategies to Evade the Host Immune Recognition. J. Fungi.

[84] Almeida-Paes R., de Oliveira L.C., Oliveira M.M.E., Gutierrez-Galhardo M.C., Nosanchuk J.D., Zancopé-Oliveira R.M. (2015). Phenotypic characteristics associated with virulence of clinical isolates from the *Sporothrix* complex. BioMed Res. Int..

[85] Alba-Fierro C.A., Pérez-Torres A., López-Romero E., Cuéllar-Cruz M., Ruiz-Baca E. (2014). Cell wall proteins of *Sporothrix schenckii* as immunoprotective agents. Rev. Iberoam. Micol..

[86] Martínez-Álvarez J.A., Pérez-García L.A., Mellado-Mojica E., López M.G., Martínez-Duncker I., Lópes-Bezerra L.M., Mora-Montes H.M. (2017). Sporothrix schenckii sensu stricto and *Sporothrix brasiliensis* Are Differentially Recognized by Human Peripheral Blood Mononuclear Cells. Front. Microbiol..

[87] Rodrigues A.M., de Hoog S., de Camargo Z.P. (2013). Emergence of pathogenicity in the *Sporothrix schenckii* complex. Med. Mycol..

[88] Rodrigues A.M., de Hoog G.S., de Cássia Pires D., Brihante R.S.N., da Costa Sidrim J.J., Gadelha M.F., Colombo A.L., de Camargo Z.P. (2014). Genetic diversity and antifungal susceptibility profiles in causative agents of sporotrichosis. BMC Infect. Dis..

[89] Camacho E., León-Navarro I., Rodríguez-Brito S., Mendoza M., Niño-Vega G.A. (2015). Molecular epidemiology of human sporotrichosis in Venezuela reveals high frequency of *Sporothrix globosa*. BMC Infect. Dis..

[90] Fischman Gompertz O., Rodrigues A.M., Fernandes G.F., Bentubo H.D.L., de Camargo Z.P., Petri V. (2016). Atypical Clinical Presentation of Sporotrichosis Caused by *Sporothrix globosa* Resistant to Itraconazole. Am. J. Trop. Med. Hyg..

[91] Alba-Fierro C.A., Pérez-Torres A., Toriello C., Romo-Lozano Y., López-Romero E., Ruiz-Baca E. (2016). Molecular Components of the *Sporothrix schenckii* Complex that Induce Immune Response. Curr. Microbiol..

[92] Carlos I.Z., Sassá M.F., da Graça Sgarbi D.B., Placeres M.C.P., Maia D.C.G. (2009). Current research on the immune response to experimental sporotrichosis. Mycopathologia.

[93] Gaze S.T., Dutra W.O., Lessa M., Lessa H., Guimarães L.H., Jesus A.R., de Carvalho L.P., Machado P., Carvalho E.M., Gollob K.J. (2006). Mucosal leishmaniasis patients display an activated inflammatory T-cell phenotype associated with a nonbalanced monocyte population. Scand. J. Immunol..

[94] Espinosa V., Rivera A. (2012). Cytokines and the regulation of fungus-specific CD4 T cell differentiation. Cytokine.

[95] Ferreira L.S., Gonçalves A.C., Portuondo D.L., Maia D.C.G., Placeres M.C.P., Batista-Duharte A., Carlos I.Z. (2015). Optimal clearance of *Sporothrix schenckii* requires an intact Th17 response in a mouse model of systemic infection. Immunobiology.

[96] Quintella L.P., Passos S.R.L., Francesconi do Vale A.C., Galhardo M.C.G., Barros M.B.D.L., Cuzzi T., Dos Santos Reis R., de Figueiredo Carvalho M.H., Zappa M.B., De Oliveira Schubach A. (2011). Histopathology of cutaneous sporotrichosis in Rio de Janeiro: A series of 119 consecutive cases. J. Cutan. Pathol..

[97] Underhill D.M., Pearlman E. (2015). Immune Interactions with Pathogenic and Commensal Fungi: A Two-Way Street. Immunity.

[98] Bär E., Whitney P.G., Moor K., e Sousa C.R., LeibundGut-Landmann S. (2014). IL-17 regulates systemic fungal immunity by controlling the functional competence of NK cells. Immunity.

[99] Gonçalves A.C., Maia D.C.G., Ferreira L.S., Monnazzi L.G.S., Alegranci P., Placeres M.C.P., Batista-Duharte A., Carlos I.Z. (2015). Involvement of major components from *Sporothrix schenckii* cell wall in the caspase-1 activation, nitric oxide and cytokines production during experimental sporotrichosis. Mycopathologia.

[100] Brown G.D. (2006). Dectin-1: A signalling non-TLR pattern-recognition receptor. Nat. Rev. Immunol..

[101] Saijo S., Iwakura Y. (2011). Dectin-1 and Dectin-2 in innate immunity against fungi. Int. Immunol..

[102] Bourgeois C., Majer O., Frohner I.E., Tierney L., Kuchler K. (2010). Fungal attacks on mammalian hosts: Pathogen elimination requires sensing and tasting. Curr. Opin. Microbiol..

[103] LeibundGut-Landmann S., Wüthrich M., Hohl T.M. (2012). Immunity to fungi. Curr. Opin. Immunol..

[104] Jellmayer J.A., Ferreira L.S., Manente F.A., Gonçalves A.C., Polesi M.C., Batista-Duharte A., Carlos I.Z. (2017). Dectin-1 expression by macrophages and related antifungal mechanisms in a murine model of *Sporothrix schenckii* sensu stricto systemic infection. Microb. Pathog..

[105] Koga T., Duan H., Furue M. (2002). Immunohistochemical detection of interferon-gamma-producing cells in granuloma formation of sporotrichosis. Med. Mycol..

[106] Koga T., Duan H., Urabe K., Furue M. (2001). Immunohistochemical localization of activated and mature CD83+ dendritic cells in granulomas of sporotrichosis. Eur. J. Dermatol..

[107] Morgado F.N., Schubach A.O., Pimentel M.I., Lyra M.R., Vasconcellos É.C.F., Valete-Rosalino C.M., Conceição-Silva F. (2016). Is There Any Difference between the In Situ and Systemic IL-10 and IFN-γ Production when Clinical Forms of Cutaneous Sporotrichosis Are Compared?. PLoS ONE.

[108] Maia D.C.G., Sassá M.F., Placeres M.C.P., Carlos I.Z. (2006). Influence of Th1/Th2 cytokines and nitric oxide in murine systemic infection induced by Sporothrix schenckii. Mycopathologia.

[109] Almeida S.R. (2012). Therapeutic monoclonal antibody for sporotrichosis. Front. Microbiol..

[110] Blanco J.L., Garcia M.E. (2008). Immune response to fungal infections. Vet. Immunol. Immunopathol..

[111] De Franco D.L., Nascimento R.C., Ferreira K.S., Almeida S.R. (2012). Antibodies against *Sporothrix schenckii* Enhance TNF-α Production and Killing by Macrophages. Scand. J. Immunol..

[112] Maia D.C.G., Gonçalves A.C., Ferreira L.S., Manente F.A., Portuondo D.L., Vellosa J.C.R., Polesi M.C., Batista-Duharte A., Carlos I.Z. (2016). Response of Cytokines and Hydrogen Peroxide to *Sporothrix schenckii* Exoantigen in Systemic Experimental Infection. Mycopathologia.

[113] Gonçalves A.C., Ferreira L.S., Manente F.A., de Faria C.M.Q.G., Polesi M.C., de Andrade C.R., Zamboni D.S., Carlos I.Z. (2017). The NLRP3 inflammasome contributes to host protection during *Sporothrix schenckii* infection. Immunology.

[114] Da Daniel Rosa W., Gezuele E., Calegari L., Goñi F. (2008). Asteroid body in sporotrichosis. Yeast viability and biological significance within the host immune response. Med. Mycol..

[115] Rodrigues A.M., Kubitschek-Barreira P.H., Fernandes G.F., de Almeida S.R., Lopes-Bezerra L.M., de Camargo Z.P. (2015). Immunoproteomic analysis reveals a convergent humoral response signature in the *Sporothrix schenckii* complex. J. Proteom..

[116] Portuondo D.L., Batista-Duharte A., Ferreira L.S., Martínez D.T., Polesi M.C., Duarte R.A., Marcos C.M., de Almeida A.M., Carlos I.Z. (2016). A cell wall protein-based vaccine candidate induce protective immune response against *Sporothrix schenckii* infection. Immunobiology.

[117] Portuondo D.L., Batista-Duharte A., Ferreira L.S., de Andrade C.R., Quinello C., Téllez-Martínez D., de Aguiar Loesch M.L., Carlos I.Z. (2017). Comparative efficacy and toxicity of two vaccine candidates against *Sporothrix schenckii* using either Montanide^TM^ Pet Gel A or aluminum hydroxide adjuvants in mice. Vaccine.

[118] Chen F., Jiang R., Wang Y., Zhu M., Zhang X., Dong S., Shi H., Wang L. (2017). Recombinant Phage Elicits Protective Immune Response against Systemic *S. globosa* Infection in Mouse Model. Sci. Rep..

[119] De Almeida J.R.F., Jannuzzi G.P., Kaihami G.H., Breda L.C.D., Ferreira K.S., de Almeida S.R. (2018). An immunoproteomic approach revealing peptides from *Sporothrix brasiliensis* that induce a cellular immune response in subcutaneous sporotrichosis. Sci. Rep..

[120] Fernandes K.S., Coelho A.L., Lopes Bezerra L.M., Barja-Fidalgo C. (2000). Virulence of *Sporothrix schenckii* conidia and yeast cells, and their susceptibility to nitric oxide. Immunology.

[121] Fernandes K.S.S., Neto E.H., Brito M.M.S., Silva J.S., Cunha F.Q., Barja-Fidalgo C. (2008). Detrimental role of endogenous nitric oxide in host defence against *Sporothrix schenckii*. Immunology.

[122] Alegranci P., de Abreu Ribeiro L.C., Ferreira L.S., de Negrini T.C., Maia D.C.G., Tansini A., Gonçalves A.C., Placeres M.C., Carlos I.Z. (2013). The predominance of alternatively activated macrophages following challenge with cell wall peptide-polysaccharide after prior infection with *Sporothrix schenckii*. Mycopathologia.

[123] Madrid I.M., Xavier M.O., Mattei A.S., Fernandes C.G., Guim T.N., Santin R., Schuch L.F., de Nobre M.O., Araújo Meireles M.C. (2010). Role of melanin in the pathogenesis of cutaneous sporotrichosis. Microbes Infect..

[124] Da Silva R.F., Bonfitto M., da Silva Junior F.I.M., de Ameida M.T.G., da Silva R.D.C. (2017). Sporotrichosis in a liver transplant patient: A case report and literature review. Med. Mycol. Case Rep..

[125] Lederer H.T., Sullivan E., Crum-Cianflone N.F. (2016). Sporotrichosis as an unusual case of osteomyelitis: A case report and review of the literature. Med. Mycol. Case Rep..

[126] Lee P.P., Lau Y.-L. (2017). Cellular and Molecular Defects Underlying Invasive Fungal Infections-Revelations from Endemic Mycoses. Front. Immunol..

[127] Almeida-Paes R., Figueiredo-Carvalho M.H.G., Brito-Santos F., Almeida-Silva F., Oliveira M.M.E., Zancopé-Oliveira R.M. (2016). Melanins Protect Sporothrix brasiliensis and *Sporothrix schenckii* from the Antifungal Effects of Terbinafine. PLoS ONE.

[128] Batista-Duharte A., Téllez-Martínez D., Aparecida Jellmayer J., Leandro Portuondo Fuentes D., Campos Polesi M., Martins Baviera A., Zeppone Carlos I. (2018). Repeated Exposition to Mercury (II) Chloride Enhances Susceptibility to *S. schenckii* sensu stricto Infection in Mice. J. Fungi.

[129] De Lima Barros M.B., Schubach A., Francesconi-do-Valle A.C., Gutierrez-Galhardo M.C., Schubach T.M.P., Conceição-Silva F., de Matos Salgueiro M., Mouta-Confort E., Reis R.S., de Fátima Madeira M. (2005). Positive Montenegro skin test among patients with sporotrichosis in Rio De Janeiro. Acta Trop..

[130] Evans K.G., Abraham R.M., Mihova D., Xu X., Frank D.M., Rosenbach M., Kim E.J. (2012). Acute onset of leg nodules in a sporotrichoid pattern—Quiz case. Diagnosis: Primary cutaneous diffuse large B-cell lymphoma, leg type (PCLBCL-LT). Arch. Dermatol..

[131] Lauermann F., Lyra M., Gaudio R. (2012). Sporotrichosis mimicking keratoacanthoma. Am. J. Trop. Med. Hyg..

[132] Nakamura S., Hashimoto Y., Nishi K., Takahashi H., Takeda K., Mizumoto T., Iizuka H. (2012). Cutaneous tuberculosis simulating lymphocutaneous sporotrichosis. Australas. J. Dermatol..

[133] Quintella L.P., Passos S.R.L., de Miranda L.H.M., Cuzzi T., Barros M.D., Francesconi-do-Vale A.C., Galhardo M.C., Madeira M.D., de Figueiredo Carvalho M.H., Schubach A.D. (2012). Proposal of a histopathological predictive rule for the differential diagnosis between American tegumentary leishmaniasis and sporotrichosis skin lesions. Br. J. Dermatol..

[134] Aung A.K., Teh B.M., McGrath C., Thompson P.J. (2013). Pulmonary sporotrichosis: Case series and systematic analysis of literature on clinico-radiological patterns and management outcomes. Med. Mycol..

[135] Di Luca D.G., De Andrade P.J.S., Sales A.M., De Menezes V.M., Galhardo M.C.G., Pimentel M.I.F., Lyra M.R., Nery J.A. (2013). Superposition of leprosy and other neglected tropical diseases in the state of Rio de Janeiro: A case series report. Lepr. Rev..

[136] Kawtar I., Salim G., Mariame M., Fatimazahra M., Imane T., Salma B., Imane E.H., Mohamed E. (2013). Sporotrichoid chromomycosis. Dermatol. Online J..

[137] Zhang Y., Pyla V. (2014). Nasal sporotrichosis in children. Int. J. Dermatol..

